# Associations Between Parent Self-Reported and Accelerometer-Measured Physical Activity and Sedentary Time in Children: Ecological Momentary Assessment Study

**DOI:** 10.2196/15458

**Published:** 2020-05-19

**Authors:** Junia N de Brito, Katie A Loth, Allan Tate, Jerica M Berge

**Affiliations:** 1 Department of Family Medicine and Community Health University of Minnesota Minneapolis, MN United States

**Keywords:** ecological momentary assessment, accelerometry, mobile devices, physical activity, sedentary behavior, children

## Abstract

**Background:**

Retrospective self-report questionnaires are the most common method for assessing physical activity (PA) and sedentary behavior (SB) in children when the use of objective assessment methods (eg, accelerometry) is cost prohibitive. However, self-report measures have limitations (eg, recall bias). The use of real-time, mobile ecological momentary assessment (EMA) has been proposed to address these shortcomings. The study findings will provide useful information for researchers interested in using EMA surveys for measuring PA and SB in children, particularly when reported by a parent or caregiver.

**Objective:**

This study aimed to examine the associations between the parent’s EMA report of their child’s PA and SB and accelerometer-measured sedentary time (ST), light-intensity PA (LPA), and moderate-to-vigorous–intensity PA (MVPA) and to examine if these associations differed by day of week, sex, and season.

**Methods:**

A total of 140 parent-child dyads (mean child age 6.4 years, SD 0.8; n=66 girls; n=21 African American; n=24 American Indian; n=25 Hispanic/Latino; n=24 Hmong; n=22 Somali; and n=24 white) participated in this study. During an 8-day period, parents reported child PA and SB via multiple daily signal contingent EMA surveys, and children wore a hip-mounted accelerometer to objectively measure ST, LPA, and MVPA. Accelerometer data was matched to the time period occurring before parent EMA-report of child PA and SB. Generalized estimating equations with interaction-term analyses were performed to determine whether the relationship between parent-EMA report of child PA and SB and accelerometer-measured ST and LPA and MVPA outcomes differed by day of the week, sex and season.

**Results:**

The parent’s EMA report of their child’s PA and SB was strongly associated with accelerometer-measured ST, LPA, and MVPA. The parent’s EMA report of their child’s PA was stronger during the weekend than on weekdays for accelerometer-measured ST (*P*≤.001) and LPA (*P*<.001). For the parent’s EMA report of their child’s SB, strong associations were observed with accelerometer-measured ST (*P*<.001), LPA (*P*=.005), and MVPA (*P*=.008). The findings related to sex-interaction terms indicated that the association between the parent-reported child’s PA via EMA and the accelerometer-measured MVPA was stronger for boys than girls (*P*=.02). The association between the parent’s EMA report of their child’s PA and SB and accelerometer-measured ST and PA was similar across seasons in this sample (all *P* values >.31).

**Conclusions:**

When the use of accelerometry-based methods is not feasible and in contexts where the parent is able to spend more proximate time observing the child’s PA and SB, the parent’s EMA report might be a superior method for measuring PA and SB in young children relative to self-report, given the EMA’s strong associations with accelerometer-measured PA and ST.

## Introduction

Reduced physical activity (PA) and increased sedentary behavior (SB) among children have been associated with less healthy body composition, reduced cardiovascular and musculoskeletal fitness, and other health problems [1]. Empirical evidence supports that important ethnic and racial disparities exist with regard to children’s engagement in PA and health outcomes associated with low levels of PA [2,3]. Owing to the importance of PA to the health of American children, the second edition of the 2008 Physical Activity Guidelines for Americans recommended that preschool-aged children (ie, aged 3-5 years) engage in active play throughout the day, and children aged 6 to 17 years accumulate at least 60 min of moderate-to-vigorous–intensity PA (MVPA) per day for disease prevention and health promotion [4]. Given these recommendations, it is of paramount importance that continued efforts are made to improve our ability to accurately assess PA and SB in children.

Several techniques have been used to assess SB and/or PA in children, including, but not limited to, doubly labeled water, accelerometry, and retrospective self-report, which have all been validated with children. Doubly labeled water is the gold standard for measuring total energy expenditure and estimating PA level; however, objective assessment of PA using this method is often cost prohibitive in large-scale epidemiologic studies. As an alternate approach, accelerometers have gained popularity as an objective measurement tool because of their feasibility of use in real-world settings and thus have become the method of choice in epidemiological studies and trials [5]. In addition, the use of accelerometers overcomes many of the recall-based limitations of retrospective assessments and provides accurate measurements of both PA and SB [6,7]. Importantly, there is evidence that the use of accelerometers is feasible with young children [8], irrespective of the unpredictable and irregular nature of play behavior [9]. However, both doubly labeled water and accelerometry techniques require considerable expertise. In addition, these methods can be costly and suffer from administrative time burden on the research team [10-12]. Retrospective self-report questionnaires are the most common method for PA and SB assessment in children and can vary by what they measure (eg, intensity, duration, and frequency of PA) [10]. Although these questionnaires are cost-effective, easy to administer, and provide good assessment of discrete categories of activity level (eg, low, moderate, and high), these questionnaires may suffer from measurement errors (ie, overestimation or underestimation of self-reported PA and SB) and rely heavily on participant’s recall ability, which may result in recall bias [13,14]. In fact, studies with children have shown that social desirability bias generally attenuates the strength of associations between reports of PA and SB with health outcomes [14,15]. In addition, previous literature showed that objectively measured PA and self-reported PA and/or PA recall assessment for children have low-to-moderate levels of agreement [16,17]. Therefore, technological advances in survey assessment of SB and PA are needed to overcome these common reporting biases. This is of particular relevance for studying children populations because young children are unlikely to provide reliable estimates of time engaged in PA [10]. Thus, researchers must rely on proxy questionnaire reports from parents and/or another adult caregiver, particularly for young children [18].

An innovative technological advance that has been introduced to minimize the limitations associated with retrospective self-reports from children is ecological momentary assessment (EMA). This technique uses real-time assessment of behavior and was developed to combine both ecological and momentary aspects of the behavior being investigated [19]. EMA is particularly suitable for studying PA and SB in children because children’s behavior can be sporadic and contextual [20]. Furthermore, studies investigating the child’s PA and SB via EMA have used different software apps and/or Web-based tools that can be loaded or prompted in a mobile device for repeated assessments throughout the day. A few studies have investigated the associations between self-reported PA and SB using EMA via mobile devices and accelerometer-measured PA and ST in children [21-23]. Specifically, Zink et al [23] reported that the use of EMA surveys by children is highly correlated with accelerometer measures, and other studies have reported EMA to be a feasible measure of PA and SB with children [21,22]. However, to our knowledge, EMA has not been investigated as a measure of a child’s PA and SB when reported by a child’s parent or other adult caregiver (hereafter *parent*). In addition, few studies using EMA have focused on populations from diverse ethnic and/or racial backgrounds and low socioeconomic status, which is important, given the health disparities in PA and SB in these populations [3]. Therefore, investigating the use of EMA for parental report of the child’s PA and SB and how well it is correlated with accelerometer-measured sedentary time (ST), light PA (LPA), and MVPA in a diverse sample is important and might be a useful option to not only overcome issues related to retrospective self-report measures by children but also for when the use of objective measurement techniques by researchers are cost prohibitive.

The purpose of this study was to investigate the associations between the parent’s report of their child’s PA and SB via electronically delivered EMA surveys and accelerometer-measured ST, LPA, and MVPA in a diverse sample and if these associations differed by day of week (ie, weekday vs weekend day), sex (ie, boys vs girls), and season (ie, summer vs school year), given that previous studies reported that children’s engagement levels in PA and SB are likely to differ by these factors [23-26]. The findings from this study might provide useful information for researchers interested in using the parent-reported, electronically delivered EMA surveys for measuring PA and SB in children.

## Methods

### Data Source

Data used in this study were drawn from phase 1 of the *Family Matters* study. The full rationale and methodology of the *Family Matters* study have been published elsewhere [27]. Briefly, phase I included in-depth mixed methods cross-sectional examinations of the home environment of racially/ethnically diverse children from low-income families (N=150). Inclusion criteria for phase 1 of the *Family Matters* study included children aged 5 to 7 years with no medical problem precluding study participation (eg, serious mental illness and disease altering diet or PA); a recent and verified medical record of BMI >5th percentile for age and sex; fluency in English, Spanish, Hmong, and/or Somali; reside with the parent participating in the study; and parent and child not currently participating in a weight management program. Participants engaged in a 10-day in-home observation period that included 2 in-home visits and an 8-day observational period in between home visits. The first home visit (baseline) included, but was not limited to, consenting procedures, demographics, anthropometric measurements (ie, weight was measured 2-3 times using a portable digital scale [Seca 869 model] and recorded to the nearest 0.1 kg; height was measured in duplicate using a portable stadiometer [Seca 217 model] and recorded to the nearest 1.0 cm), and EMA and accelerometry training. The present analyses used selected demographic and anthropometric variables and child accelerometry and EMA data from phase 1. The University of Minnesota Institutional Review Board approved phase 1 of the *Family Matters* study, and all participants consented in accordance with the Declaration of Helsinki procedures [28].

### Participants

The analytic sample of this study included 140 parent-child dyads, with children aged 5 to 7 years from 6 racial and ethnic groups (African American, n=21; American Indian, n=24; Hispanic/Latino, n=25; Hmong, n=24; Somali, n=22; and white, n=24). Ten children were excluded because they did not meet the *Family Matters* study protocol for daily accelerometer wear time (ie, 8 hours for 3 weekdays and 1 weekend day). The demographic and anthropometric characteristics of these participants were similar to the full sample population (N=150). On average, the children included in this sample were aged 6.4 (SD 0.8) years, 47.1% were girls (66/140), and 47.8% (67/140) were overweight or obese (BMI percentile ≥85). Parent-child dyads were, on average, primarily low income (98/140, 70.0% with household income <US $34,000) and comprised of young adult parents (129/140, 92.1% female, 11/140, 7.9% male; mean age 35 years, SD 7.1) who worked full time (62/140, 44.3%) and had at least a high-school education (105/140, 75.0%).

### Measures

#### Accelerometer

Children’s PA levels and ST were assessed objectively using a duo-dimensional accelerometer (ActiGraph GT1M; ActiGraph, LLC), which has been previously validated with children [6,24]. The GT1M devices sampled activity in 15-second epochs, which were then processed to determine ST, LPA, and MVPA duration, as determined by Evenson et al [29]. ST corresponded to 0 to 100 counts per min, LPA corresponded to 101 to 2295 counts per min, and MVPA corresponded to 2296 or more counts per min. Accelerometer nonwear time criteria were defined as more than 60 consecutive min of zero counts, and nonwear and nonvalid data were removed before analysis. For this study, a minimum wear time criterion was determined to be 4 days (ie, 3 weekdays and 1 weekend day) over the 8-day observation period, with at least four waking hours of wear time per day.

Participants were instructed to wear the accelerometer for all waking hours during 8 consecutive days and to only remove it for sleeping, bathing, and/or water-based activities (eg, swimming). Accelerometers were attached to an elastic belt and fitted on the child’s right hip, with the parent’s supervision to ensure proper placement. Detailed written and verbal instructions regarding accelerometer wear were provided for both children and parents to facilitate compliance. Families were compensated with a US $25 gift card, in addition to the study incentive of approximately US $300 for complying with the full accelerometry wear-time demands.

### Ecological Momentary Assessment

The parent’s report of their child’s PA and SB was measured via multiple daily signal-contingent EMA surveys [30] over an 8-day observation period. More specifically, a minimum of two of 4 signal-contingent daily surveys per day was necessary to be considered a valid response day. Parents were provided an iPad (Apple Inc) and received verbal and written instructions during the first in-home visit, in addition to hands-on training on how to use this mobile device to respond to EMA surveys. Signal-contingent EMA recordings were prompted with a beep on the iPad or via text message as a start signal and were programmed to be delivered randomly, 4 times a day, within an interval of 3-hour time block, from 7 to 10 am, 12 to 2 pm, 3 to 6 pm, and 7 to 10 pm. The EMA survey link expired after 1 hour. In addition, the EMA surveys were delivered in the parents’ preferred language (ie, English, Spanish, Somali, or Hmong), and the scheduled EMA prompt delivery was adjusted for parent shift work and wake times to accommodate parents’ differing life circumstances.

In this study, the following EMA questions were used to examine the parent’s report of their child’s PA and SB: (1) *Since the last survey/Since you woke up this morning has [child’s name] done something physically active?* (yes/no) and (2) *Since the last survey/Since you woke up this morning has [child’s name] watched television/movies or played video games*? (yes/no), respectively. The former was used as a proxy measure for the parent’s understanding of child engagement in LPA and MVPA, and the latter was used as a proxy measure for the parent’s understanding of child engagement in SB. To facilitate compliance, participants were asked to carry the iPad with them throughout the day. Parents were not required to be in the presence of their child when answering signal-contingent EMA survey questions. Other requirements and details regarding the EMA surveys used in the *Family Matters* study have been published elsewhere [27].

### Statistical Analysis

The child’s PA and ST were measured at the hour level using an accelerometer. These data were matched to the period occurring before the parent’s EMA assessment of their child’s PA and SB on the same observation day and period of accelerometer wear time. For example, if an EMA survey was completed at any time during the 10 am hour of a Monday, accelerometer time was matched to that EMA survey report through 9:59 am of that same Monday to ensure the retrospective assessment did not include accelerometer time measured after the parent EMA survey report. Cross-tabulations and descriptive statistics were used to identify patterns of wear time, ST, LPA, and MVPA over the course of the day. All outcome variables were continuous (ie, minutes of ST, LPA, and MVPA) and were standardized to accelerometer minutes per hour measured in the signal-contingent EMA survey pre-period. The primary predictor variable was dummy-coded parent’s report of the child’s engagement in SB and PA before the EMA survey assessment.

The generalized estimating equation approach was used as the primary analytical method. Huber/White robust standard errors were used to deal with model misspecification and correlated participant error terms because of repeated measures [31]. A Gaussian variance family with identity link was used, and a within-participant correlation structure was set to independent to preserve statistical precision [32]. Residual versus fitted plots were used to examine model fit, and any patterns in the error term by predicted mean level. Adjusted models included child race/ethnicity, normal weight/overweight status, day of the week, sex, age, season, and household income. Interaction-term analyses were also performed to determine if the relationships between the parent’s EMA report of their child’s PA and SB and accelerometer-measured ST, LPA, and MVPA outcomes were modified by day of the week (ie, weekday vs weekend day), sex (ie, boys vs girls), and season (ie, summer vs school year). The interaction terms between the parent’s EMA report of their child’s PA or SB with day of the week, sex, and season were included separately in each model. Adjusted Wald chi-square tests assessed if EMA compliance and accelerometry wear time differed by day of week, child sex, and season. Sensitivity analyses were performed to determine if more strict accelerometer wear time inclusion criteria (≥8 hours) affected the direction and magnitude of association of our findings, and the results were not affected. The 4-hour minimum inclusion criterion was retained for all models. All data management and analyses were performed in Stata 15.1 MP (StataCorp).

## Results

### Ecological Momentary Assessment and Accelerometry Compliance

Of 150 parent-child dyads, 140 complied with the minimum EMA and accelerometry requirements. In total, 3127 EMA surveys were completed and successfully matched to 944 days of eligible accelerometer data. Overall, compliance (ie, minimum of two surveys completed out of total number of surveys completed) of daily signal-contingent EMA surveys was 82.8% (3127/3776), and 88.5% (124/140) of respondents completed at least three or more surveys, indicating high EMA survey engagement. In addition, in the analytic sample, the average child accelerometer wear time was 7.8 hours per day. Neither EMA compliance nor accelerometry wear time differed by day of week, child sex, or season.

### During What Periods Are Children More or Less Physically Active?

Table 1 descriptively shows average hours of child accelerometer wear time as well as minutes per hour of ST, LPA, and MVPA by order and time of EMA survey completion. On average, children wore the accelerometer for approximately 2 hours before the time in which parents answered the EMA survey, with 71% (5.5/7.8 hours) of the wear time encompassing the first 2 daily EMA surveys. Before the first survey, children spent, on average, two-thirds of an hour engaged in ST (38 min per hour), about one-third of an hour in LPA (19 min per hour), and 3 min per hour in MVPA. As the day progressed, less time was spent in ST, and more time was spent in LPA, with modest increases in MVPA. Notably, in between the third and fourth EMA survey answered, children spent half of their time engaged in ST (30 min per hour), 25 min per hour in LPA, and 5 min per hour in MVPA.

**Table 1:** Accelerometer-measured average minutes of the child’s physical activity and the composition of physical activity category by the time of ecological momentary assessment survey completion (N=140 for respondents; N=944 for observation days; and N=3127 for ecological momentary assessment surveys).

Daily order of EMA^a^ survey	Average time of EMA survey completion	Accelerometer physical activity category measured before EMA survey completion			
		Average child wear time (hours)	Sedentary time, minutes/hour (wear time), n (%)	Light-intensity physical activity, minutes per hour (wear time), n (%)	Moderate-to-vigorous–intensity physical activity, minutes per hour (wear time), n (%)
First survey	9:56 AM	2.1	38 (64)	19 (32)	3 (4)
Second survey	2:04 PM	3.4	32 (53)	24 (40)	4 (7)
Third survey	4:02 PM	1.7	30 (50)	25 (42)	5 (8)
Fourth survey	5:27 PM	0.6	30 (5)	25 (42)	5 (8)

^a^EMA: ecological momentary assessment.

### Did Day of the Week Modify the Association Between Parent Ecological Momentary Assessment Report of the Child’s Physical Activity and Sedentary Time and the Accelerometer-Measured Sedentary Time, Light-Intensity Physical Activity, and Moderate-to-Vigorous–Intensity Physical Activity?

The average stratum estimates of ST, LPA, and MVPA for day of the week statistical interaction analyses when the parent reported/did not report the child’s PA and/or SB are presented in the Multimedia Appendix 1. The results indicated that on weekends relative to weekdays, the parent’s report of their child’s PA via EMA was more strongly, negatively associated with accelerometer-measured ST (*P*_interaction_≤.001) and positively associated with LPA (*P*_interaction_<.001), but with similar levels of MVPA (*P*_interaction_=.37) during weekends and weekdays. Notably, on weekends, the parent’s EMA report of their child’s PA was associated with an average decrease of −5.1 (95% CI −6.9 to −3.3) min per hour of ST and an average increase of 4 (95% CI 2.4 to 5.5) min per hour in LPA and 1.1 (95% CI 0.3 to 1.9) min per hour in MVPA, relative to the parent’s EMA report of their child not engaging in PA. The results also indicated that on weekends, the parent’s EMA report of their child’s SB was strongly positively associated with accelerometer-measured ST (*P*_interaction_<.001) and negatively associated with both LPA and MVPA (*P*_interaction_=.005 and .008, respectively). The relationship between the parent’s EMA report of their child’s SB and accelerometer-measured ST and LPA was stronger on weekends than on weekdays. On weekends, the parent’s EMA report of their child’s SB was associated with average increase of 2.8 (95% CI 0.9 to 4.7) min per hour of ST and average decrease of −1.6 (95% CI −3.23 to 0.03) min per hour of LPA and −1.2 (95% CI −2.1 to −0.4) min per hour of MVPA relative to the parent’s EMA report of their child not engaging in SB.

### Did Sex Modify the Association Between Parent Ecological Momentary Assessment Report of the Child’s Physical Activity and Sedentary Behavior and the Accelerometer-Measured Sedentary Time, Light-Intensity Physical Activity, and Moderate-to-Vigorous–Intensity Physical Activity?

The results indicated that the association between the parent’s report of their child’s PA and accelerometer-measured MVPA differed between boys and girls (*P*_interaction_=.02) in this sample (see Multimedia Appendix 2).

Specifically, for boys, the parent’s EMA report of their child’s PA was associated with an average increase of 1.2 (95% CI 0.7 to 1.7) min of MVPA per hour compared with when the parent reported the child was not engaging in PA. For girls, our findings revealed similar amounts of average minutes per hour of MVPA (0.2; 95% CI −0.5 to 0.9) when parents reported the child’s PA relative to when they did not report the child’s PA via EMA. The statistical interaction analyses for the parent’s EMA report of their child’s PA and accelerometer-measured ST and LPA association revealed similar results between boys and girls (*P*_interaction_=.17 and .55, respectively). In addition, our results indicated that the association between the parent’s EMA report of their child’s SB and accelerometer-measured ST and LPA somewhat differed between boys and girls (*P*_interaction_=.049 and .05, respectively) in this sample. For boys, the results showed that when the parent reported the child’s SB via EMA, it was associated with an average increase of 1.4 (95% CI −0.2 to 3.0) min per hour of ST and an average decrease of −0.9 (95% CI −2.2 to 0.4) min per hour of LPA, relative to when the parent reported the child not engaged in SB. For girls, however, our results revealed that when the parent reported the child engaged in SB via EMA, it was associated with an average decrease of −1.1 (95% CI −3.0 to 0.8) min per hour of ST and an average increase of 1.2 (95% CI −0.4 to 2.9) min per hour of LPA, relative to when the parent reported the child not engaged in SB. According to our statistical interaction analysis, when the parent reported the child engaged in SB, boys and girls had similar levels of MVPA (*P*_interaction_=.39).

### Did Season Modify the Association Between Parent Ecological Momentary Assessment Report of the Child’s Physical Activity and Sedentary Behavior and the Accelerometer-Measured Sedentary Time, Light-Intensity Physical Activity, and Moderate-to-Vigorous–Intensity Physical Activity?

The statistical interaction analysis did not indicate that the association between the parent’s EMA report of their child’s PA and accelerometer-measured ST, LPA, and MVPA differed by season (ST: *P*_interaction_=.41; LPA: *P*_interaction_=.43; and MVPA: *P*_interaction_=.59) in this sample. Similarly, regarding the association between the parent’s EMA report of their child’s SB and accelerometer-measured ST, LPA, and MVPA, the results of the interaction analysis revealed similar results across seasons (ST: *P*_interaction_=.85; LPA: *P*_interaction_=.89; and MVPA: *P*_interaction_=.31).

## Discussion

### Principal Findings

This study examined the relationships between the parent’s report of their child’s PA and SB via electronically delivered EMA surveys and simultaneous objective (ie, accelerometry) measurement of child engagement in minutes per hour of ST and PA (ie, LPA and MVPA) and if these associations differed by day of the week, sex, and season. The results revealed that the use of mobile EMA surveys by parents to report the child’s PA and SB was strongly associated with accelerometer-measured ST, LPA, and MVPA in this sample. Day of the week and sex were identified as moderators of these relationships. These findings suggest that the parent-reported EMA surveys might be a suitable measure for capturing PA and SB in children, particularly when the parent is able to spend more proximate time observing the child’s sedentary and PA behaviors.

Our results showed a stronger association between the parent’s EMA report of their child’s PA and SB and accelerometer-measured ST, LPA, and MVPA and ST and LPA, respectively, for weekend days than on weekdays. These results are consistent with other studies [24,26,33]. Given that the majority of our sample included parents who worked full time, it is plausible that parents were more often in the presence of their child during weekends and, therefore, were able to provide better estimates of their child’s PA and SB during weekend days relative to weekdays via EMA. Previous research indicated that retrospective, parent self-reported PA and SB methods are not a suitable proxy measure for assessing 2- to 9-year-old children’s PA and SB [34]. In fact, these retrospective methods rely on the respondent’s memory, which can lead to recall bias [35]. Given that mobile technology is now widely available, researchers may rely on electronically delivered EMA surveys to overcome these self-report issues related to more traditional retrospective assessments (eg, 7-day physical activity recall). Therefore, EMA might be advantageous over retrospective survey methods because EMA surveys can be programmed to be delivered multiple times during the day, with such prompting frequency that might facilitate understanding of daily life health behaviors, reduce recall bias if answered promptly, and improve ecological validity and generalizability. Future research using the parent-reported EMA surveys to capture PA and SB of children might use these results to better program their EMA prompts and to also anticipate when parents are more likely to provide more accurate reports of the child’s PA and SB. For instance, these studies could incorporate more EMA prompts for the times in which the parent is with the child (eg, evenings hours and weekends) and also incorporate in the study protocol EMA prompts that could be delivered directly to the child, which could be particularly a good strategy for older children.

Sex differences in parent EMA-reported PA and SB and accelerometer-measured minutes of PA and ST were also observed in this sample. Specifically, our results showed a stronger association for boys relative to girls for the relationship between parent EMA-reported PA and accelerometer-measured MVPA. This finding is consistent with previous studies using both objective and self-reported measurements of children’s PA, in which showed that boys generally engage in more minutes of MVPA than girls [36-39]. In addition, our results noting sex differences for the association between parent EMA-report of SB and accelerometer-measured ST and LPA are aligned with numerous other studies [23,33,40,41]. It is noteworthy to mention that we observed an inverse association between boys and girls for the association between parent EMA-reported SB and accelerometer-measured ST and LPA. It is possible that parents in our sample perceived boys more often engaged in SB activities (eg, playing video game), whereas girls more often engaged in more LPA (eg, dancing) activities. These findings might be useful for future studies using EMA as a measurement instrument of PA and SB for two main reasons. First, EMA survey question and answer selection could be appropriately designed to reflect specific SB and LPA that are often preferred or habitually practiced by boys (eg, playing video game) and girls (eg, craft, playing musical instruments) [42-44]. Second, these studies could also incorporate children’s specific activities preferences for school time, recess time, and leisure time as a way to better capture specific sedentary and LPA behaviors [45,46].

The season has also been noted to be an important factor in modifying children’s PA behaviors [25]. However, our results stand in contrast to previous studies examining season effects on the child’s PA and SB [47,48], as the association between the parent EMA-reported child’s PA and SB and accelerometer-measured ST, LPA, and MVPA remained similar across seasons in our sample. A potential explanation as to why our results differed from past studies may be because of the fact that season was defined differently across studies. For example, some studies defined it as fall versus spring [47], whereas others defined it as warmer versus colder months [48]. This inconsistency in seasonal characterization makes it challenging to compare findings across studies. Therefore, future studies investigating season effects on PA and SB levels in children might benefit from employing more rigorous characterization of seasonality (eg, weather, ecology, hours of daylight, and geographic region).

### Strengths and Limitations

This study has several strengths, including (1) the use of electronically delivered EMA surveys to measure the child’s PA and SB, with a flexible prompting frequency delivered repeatedly at different waking hours within an 8-day observation period, which reduced reliance on participant’s memory thus reducing the likelihood of recall bias; (2) the use of objective measurement of PA and SB via accelerometry, which is particularly suitable for measuring PA and ST; (3) data collection spanning different seasons and all months of the year; and (4) the inclusion of racially/ethnically and socioeconomically diverse participants, as well as immigrant populations, which increases generalizability within vulnerable/high-risk populations. However, some limitations are noteworthy. First, the dichotomous nature of the EMA survey questions about PA and SB and the lack of clearer descriptions of what constitutes LPA (eg, standing and walking slowly), MVPA (eg, running and biking fast), and SB (eg, time spent watching television or video game) might have led parents to interpret PA and SB differently, which could have resulted in some measurement error. Future studies using EMA surveys might benefit from providing clearer descriptions of what constitutes LPA, MVPA, and SB in their questions, specifically those associated with childhood obesity and other preventable health conditions (eg, viewing television/movie, playing computer and video games, or other screen-related SBs) [49,50]. This would allow parents to provide better reports of their child’s behaviors, thus allowing researchers to use more accurate information regarding the specific types of behaviors, such as SBs among children that could be targeted in future behavior intervention trials. Second, it was unknown if parents were in the presence of their child while answering the EMA surveys. Therefore, it is possible that parents were not always observing their child when prompted to answer the EMA surveys and were instead making an educated guess regarding their child’s sedentary and PA behaviors, likely because they are knowledgeable about their child’s routine [51]. In addition, given that self-report methods of PA (either reported by older children or parent proxy for younger children) might be prone to social desirability bias, we cannot rule out this possibility in our sample. Third, given that accelerometer wear time decreased considerably later in the day and around the time that the fourth signal-contingent EMA survey was delivered to the parent, it is possible that accelerometer-measured child ST, LPA, and MVPA were underestimated during that time of the day. Fourth, our accelerometer wear time data were summarized to the hour, and although responses were uniformly assessed within the hour, future studies should attempt to collect and summarize accelerometer data to at least the minute to gain more precise estimates of PA and ST. Fifth, our results may not be generalizable to activity, and SBs performed outside the times in which the signal-contingent EMA prompts were answered by the parents.

### Conclusions

Our findings indicated that the parent’s report of their child’s PA and SB via electronically delivered EMA surveys were strongly associated with accelerometer-measured ST, LPA, and MVPA in children aged 5 to 7 years. Notably, these associations were stronger during weekend days than on weekdays. In addition, the parent’s EMA report of PA and accelerometer-measured MVPA were more strongly associated in boys relative to girls. The association between the parent’s EMA report of SB and accelerometer-measured ST and LPA also differed between boys and girls. Given these findings, in contexts where the parent is able to spend more proximate time observing the child’s engagement in PA and SB, the parent’s EMA report of their child’s PA and SB might be a useful and cost-effective method for measuring PA and SB, particularly in young children and relative to other retrospective self-report measures. Although the concomitant use of EMA and accelerometry is recommended, the use of mobile EMA surveys could be considered when the use of objective measurement of ST and PA are cost prohibitive.

## Appendix

Multimedia Appendix 1 Association between ecological momentary assessment of the parent-reported child’s physical activity and sedentary behavior on accelerometer-measured minutes of sedentary time, light physical activity, and moderate-to-vigorous physical activity per hour in the ecological momentary assessment survey pre-period by day of the week (N=140 for matched ecological momentary assessment accelerometry respondents; N=944 for observation days; and N=3127 for ecological momentary assessment surveys).

Multimedia Appendix 2 Association between ecological momentary assessment of the parent-reported child’s physical activity and sedentary behavior on accelerometer-measured minutes of sedentary time and light and moderate-to-vigorous physical activities per hour in the ecological momentary assessment survey pre-period by sex (N=140 for matched ecological momentary assessment accelerometry respondents; N=944 for observation days; N=3127 for ecological momentary assessment surveys).

## Abbreviations

EMA: ecological momentary assessment
LPA: light-intensity physical activity
MVPA: moderate-to-vigorous–intensity physical activity
PA: physical activity
SB: sedentary behavior
ST: sedentary time
