# Supplementary material for: Associations Between Parent Self-Reported and Accelerometer-Measured Physical Activity and Sedentary Time in Children: Ecological Momentary Assessment Study
Source: JMIR Mhealth Uhealth. 2020 May 19;8(5):e15458. doi: 10.2196/15458 (PMC7267997; doi:10.2196/15458)
Supplement: Multimedia Appendix 1 [file mhealth_v8i5e15458_app1.docx]

**Multimedia Appendix 1.** Association between ecological momentary assessment of the parent-reported child’s physical activity and sedentary behavior on accelerometer-measured minutes of sedentary time, light physical activity, and moderate-to-vigorous physical activity per hour in the ecological momentary assessment survey pre-period by day of the week (N=140 for matched ecological momentary assessment accelerometry respondents; N=944 for observation days; and N=3127 for ecological momentary assessment surveys).

| Ecological momentary assessment variable^a^ | | | Accelerometer-measured average minutes by physical activity category (minutes per hour) | | | | | | | | | | | |
| --- | --- | --- | --- | --- | --- | --- | --- | --- | --- | --- | --- | --- | --- | --- |
|  |  |  | Sedentary time | | | Light physical activity | | | | Moderate-to-vigorous physical activity | | | |  |
|  |  |  | Mean (95% CI) | Stratum mean difference | *P* value | Mean (95% CI) | Stratum mean difference | *P* value | | Mean (95% CI) | Stratum mean difference | *P* value | |  |
| **Parents’ report of their child’s physical activity (yes/no)** | | | | | | | | | | | | |  | |
|  | **Weekend** | | | | | | | | | | | | | |
|  |  | Yes | 29.8 (28.5 to 31.3) | -5.1 (-6.9 to -3.3) | <.001 | 25.3 (24.1 to 26.4) | 4 (2.4 to 5.5) | <.001 | | 4.9 (4.4 to 5.5) | 1.1 (0.3 to 1.9) | .009 | |  |
|  |  | No | 34.9 (33.4 to 36.3) | N/A^b^ | N/A | 21.3 (20 to 22.6) | N/A | N/A | | 3.9 (3.3 to 4.5) | N/A | N/A | |  |
|  | **Weekday** | | | | | | | | | | | | | |
|  |  | Yes | 31.5 (30.2 to 32.6) | -1.2 (-2.6 to 0.2) | .10 | 24 (23.1 to 24.9) | 0.5 (-0.6 to 1.6) | 0.35 | | 4.4 (4.1 to 4.8) | 0.7 (0.2 to 1.2) | .009 | |  |
|  |  | No | 32.7 (31.7 to 33.7) | N/A | N/A | 23.5 (22.7 to 24.4) | N/A | N/A | | 3.8 (3.4 to 4.2) | N/A | N/A | |  |
|  | *P*^c^ | | ≤.001 | N/A | N/A | N/A | <.001 | N/A | | N/A | .37 | N/A | |  |
| **Parents’ report of their child’s sedentary behavior (yes/no)** | | | | | | | | | | | | |  | |
|  | **Weekend** | | | | | | | | | | | | | |
|  |  | Yes | 33.6 (32.1 to 35.1) | 2.8 (0.9 to 4.7) | .004 | 22.6 (21.3 to 23.8) | -1.6 (-3.2 to 0.03) | | .05 | 3.8 (3.3 to 4.4) | -1.2 (-2.1 to -0.4) | .006 | |  |
|  |  | No | 30.8 (29.4 to 32.2) | N/A | N/A | 24.2 (22.9 to 25.4) | N/A | | N/A | 5 (4.4 to 5.7) | N/A | N/A | |  |
|  | **Weekday** | | | | | | | | | | | | | |
|  |  | Yes | 31.7 (30.6 to 32.7) | -0.9 (-2.2 to 0.4) | .16 | 24.2 (23.3 to 25.1) | 0.8 (-0.3 to 1.9) | | .15 | 4.1 (3.7 to 4.6) | 0.1 (-0.4 to 0.6) | .56 | |  |
|  |  | No | 32.6 (31.6 to 33.6) | N/A | N/A | 23.4 (22.6 to 24.3) | N/A | N/A | | 4 (3.6 to 4.3) | N/A | N/A | |  |
|  | *P*^c^ | | <.001 | N/A | N/A | N/A | .005 | N/A | | N/A | .008 | N/A | |  |

^a^Models adjusted for child race, age, sex, weight status, season, and household income. Interaction terms of the parent’s report of their child’s physical activity or sedentary behavior with day of week (weekend vs weekday) variable were included in each model separately.

^b^N/A: not applicable.

^c^*P*: *P* value for interaction.
